# Enhancement of Refractive Index Sensitivity Using Small Footprint S-Shaped Double-Spiral Resonators for Biosensing

**DOI:** 10.3390/s23136177

**Published:** 2023-07-05

**Authors:** Anh Igarashi, Maho Abe, Shigeki Kuroiwa, Keishi Ohashi, Hirohito Yamada

**Affiliations:** 1 Graduate School of Engineering, Tohoku University, Sendai 980-8579, Japan; 2 Research Institute of Electrical Communication, Tohoku University, Sendai 980-8577, Japan; 3 R&D Group, KOKOROMI Inc., Shinjuku-ku, Tokyo 169-0051, Japan; kuroiwa@kokoromill.com (S.K.);

**Keywords:** refractive index, ring resonator, evanescent field sensing, cavity length

## Abstract

We demonstrate an S-shaped double-spiral microresonator (DSR) for detecting small volumes of analytes, such as liquids or gases, penetrating a microfluidic channel. Optical-ring resonators have been applied as label-free and high-sensitivity biosensors by using an evanescent field for sensing the refractive index of analytes. Enlarging the ring resonator size is a solution for amplifying the interactions between the evanescent field and biomolecules to obtain a higher refractive index sensitivity of the attached analytes. However, it requires a large platform of a hundred square millimeters, and 99% of the cavity area would not involve evanescent field sensing. In this report, we demonstrate the novel design of a Si-based S-shaped double-spiral resonator on a silicon-on-insulator substrate for which the cavity size was 41.6 µm × 88.4 µm. The proposed resonator footprint was reduced by 680 times compared to a microring resonator with the same cavity area. The fabricated resonator exposed more sensitive optical characteristics for refractive index biosensing thanks to the enhanced contact interface by a long cavity length of DSR structures. High quality factors of 1.8 × 10^4^ were demonstrated for 1.2 mm length DSR structures, which were more than two times higher than the quality factors of microring resonators. A bulk sensitivity of 1410 nm/RIU was calculated for detecting 1 µL IPA solutions inside a 200 µm wide microchannel by using the DSR cavity, which had more than a 10-fold higher sensitivity than the sensitivity of the microring resonators. A DSR device was also used for the detection of 100 ppm acetone gas inside a closed bottle.

## 1. Introduction

In recent years, biosensors with several requirements of label-free detection, a fast response, a low cost, a high sensitivity, and a compact size measurement for use in the development of healthcare diagnoses and environmental monitoring have received increasing attention [1,2,3,4,5,6]. Thus far, enzyme-linked immunosorbent assay (ELISA) detection has been the gold-standard labeled immunoassay for detecting biomarkers, including antibodies, antigens, and proteins, with an ultralow detection limit of about 1 pM [7]. ELISA can provide a high specificity and sensitivity because of specific antigen–antibody reactions. However, the ELISA method needs time and additional costs with a high-level laboratory and well-trained specialists due to the difficulties in label-based measurements. A simple label-free and real-time detection method has become increasingly attractive. Optical biosensors, based on the interaction between the evanescent wave and particles, are being developed as sensitive and label-free quantification measurement systems [5,6,8]. The evanescent field is defined as the radiation close to the interface of two refractive media of different indices, and the radiation intensity decreases exponentially with the distance from the boundary. The biosensing mechanism is based on the sensitive interaction between the field and the attached biomolecules that have different refractive indices in the range of the evanescence field height, or penetration depth [9,10]. The refractive index can be used to determine an object, and it has a correlation with the concentration of the particles inside a substance. The detection of a very small amount of changed refractive index has been a target in the development of label-free biosensors using evanescent-wave-based optical sensing [11,12,13,14,15]. Surface plasmon resonance (SPR) has attracted much interest as a label-free biomolecular detection method with a high sensitivity in real time based on measuring the angle, wavelength, and intensity corresponding to a change in the refractive index of the medium near the metal surface [11,15,16,17,18]. However, the SPR-based biosensors remain a challenge in system miniaturization and high-cost settings [19].

Meanwhile, silicon photonics has increasingly attracted attention due to the combination of biosensing together with the development of the fabrication process for photonic-integrated circuits. Several promising candidates, such as Mach–Zehnder interferometers (MZIs), Bragg gratings, and microring resonators (MRRs), can quantitatively recognize biomolecules that have different refractive indices [2,3,12,20,21]. Based on the structure having a high contrast between the refractive index of the core layer of silicon and that of a cladding layer of silicon dioxide, silicon nitride, and the media surrounding the sensor surface, silicon photonic sensors impress with their simple structures, reusable devices, and multiplexed biomolecular detectors [1,22]. Among these methods, a ring-resonator-based method has been adopted as a simple, multiplexed-array-compatible, and sensitive biosensor that is capable of detecting a precise resonance wavelength shift as a product of the interaction between attached biomolecules and the electric field. A ring resonator used with microfluidic channels for detecting a small-volume solution and its integration with a CMOS electronic device as an all-in-one chip is highly attractive [5,22,23,24]. In addition, optical biosensors using ring resonators have increasingly become more reliable owing to the modification of receptor activities or functionalization of the biorecognition materials on the sensing surface, for example by using aptamer molecules to increase the binding events [8]. By these means, a high-sensitivity and -selectivity optical biosensor can be obtained by two methods: amplifying the output signals by increasing the interaction intensity and density using a modified-structural resonator within the penetration depth, and modifying the sensing surface using optimized surface materials [4,25,26]. There are several impactful applications for cancer diagnoses of multiplexing nanophotonic biosensors, the detection of aptamer thin layers, and the monitoring of the hemoglobin concentration in the human body [13,27,28,29]. A silicon photonics waveguide-array sensor with polydimethylsiloxane polymer cladding has been reported for applications in the detection of volatile organic compounds [30]. This research paper focuses on the improvement of the sensitivity by the method of amplifying the output signals.

Si microring resonator (MRR) biosensors are a potential candidate to achieve a compact-size, simple-fabrication, and high-sensitivity detection method [8]. The evanescence field, which is formed from the high contrast between the Si core layer of the waveguides and the cladding layers, interacts with different attached biomolecules, which pursue different refractive indices, to elevate the change in resonance wavelengths caused by the microring ring cavity [5,23]. MRRs only detect attached biomolecules within the evanescent field of several nanometers or the side wall of the cavity. This property can produce a high-sensitivity optical biosensor as most of the target molecules bind to the sensing surface. Enlarging the sensing surface or increasing the interaction between the evanescent field and the analyte is a method for amplifying the optical output signals to achieve an ultra-sensitivity [26,31]. However, an enlarged microring resonator is not a suitable approach for sensing a small-volume solution within a microfluidic channel because the enlarged cavity requires a large platform of a hundred square millimeters. Utilizing a narrow microchannel for small-volume detection becomes a chip miniaturization issue. Moreover, a large portion of biomolecules within the height of the evanescent field, or the penetration depth, cannot be involved in the sensing activities. The conventional MRR structure which has a ring resonator with a radius of several hundred micrometers might achieve an impact sensitivity; however, only a small part of the biomolecules in the analyte solution can immobilize on the sensor surface and interact with the evanescent field, while the other biomolecules will be free and will pass over the surface.

In this study, we demonstrate a design of a silicon S-shaped double-spiral resonator (DSR) with a compact platform to increase the refractive index sensitivity for detecting a small amount of the analytes in a narrow microchannel. The cavity comprises two spiral microresonators on a silicon-on-insulator (SOI) substrate, which are connected to each other by an S-shaped channel. Two impact effects of the DSR cavity are demonstrated. Firstly, the DSR structure allows the cavity length to be extended in a compact size. The extended-length cavity increases the attached biomolecules onto the area of contact interface of the resonator to generate more interaction between the electric field and biomolecules within the penetration depth, thus achieving impactful changes in the output signal’s quality factor and bulk sensitivity [31,32]. Secondly, the structure of the DSR cavity supports an easy fabrication and the alignment with a narrow microfluidic channel formed over the device. To achieve the goal of measuring a small-volume solution using a microchannel, the ideal microchannel width is approximately between 100 and 200 µm. Therefore, minimizing the width of the spiral resonator is a method to fit the cavity in the narrow microchannel easily by the naked eye. Based on these effects, the research on the optical characteristics and the sensitivity of the DSR cavity is meaningful in the development of evanescence-based biosensors.

To modify the DSR structure, simulations using the finite-difference time-domain (FDTD) method were mainly utilized to analyze the resonance characteristics for optimizing the structural parameters, such as the waveguide width, the spacing distance between turns, the curvature radius, and the gap with a bus waveguide. DSR structures were compared regarding their cavity lengths, optical characteristics, and sensitivities in simulation. Several DSR cavities were experimentally fabricated using microfabrication processes based on the simulated structures. The optical transmission spectra, measured using a custom measurement system, were collected to analyze the resonance characteristics. Detection in IPA with different concentrations from 0.1% to 0.4% was demonstrated with 1 µL solutions within the microfluidic channel to investigate the sensitivity. Finally, we examined the resonance wavelength change in response to the presence of acetone vapor in a closed bottle. In this research, IPA and acetone were selected to prove the biosensing performance of a small amount of the analytes, particularly the volatile organic compounds (VOCs). VOCs exist in the human body, and out-of-range concentrations can cause harmful effects to human health. VOCs are also known as biomarkers to discover diseases at an early stage [33,34]. Monitoring a low concentration of VOCs in air and solution with a simple real-time measurement system has been well studied [35,36,37]. IPA and acetone are popular VOCs with high vapor pressure and evaporate quickly. A simple experimental setting using the DSR-based device was used to examine the sensitivity for detecting small-volume analytes in gas and liquid environments.

## 2. Sensing Principle

The S-shaped DSR has two single Archimedean spirals (left and right) and an S-shaped channel. DSR biosensors detect attached biomolecules with different values of refractive index (RI), which are based on the interactions between the evanescent field formed over the waveguide of the resonator and the analytes. The input light from a tunable laser is coupled to a bus waveguide through a taper-edged coupler. A part of the light is subsequently coupled to the DSR cavity when the light wavelengths satisfy the resonance condition of

(1) $$\lambda_{res}=\frac{n_{eff}L}{m}$$

where m is an integer, $\lambda_{res}$ is the resonance wavelength, $n_{eff}$ is the effective refractive index, and L is the length of the DSR cavity [38]. When the resonance occurs, the resonance wavelengths can be detected as dip wavelengths in the transmission spectrum of the output light. The effective refractive index $n_{eff}$ is determined by the refractive indices of the core material and the cladding layer. At the time that the biomolecules attach to the surface of the DSR cavity, the effective index of the cavity changes owing to the interactions between the electrical field and the biomolecules, introducing a shift in the resonance wavelength $\Delta \lambda_{res}$:

(2) $$\Delta \lambda_{res}=\Delta n_{eff}\times \frac{\lambda_{res}}{n_{g}}$$

The effective group index of the cavity waveguide ($n_{g}$) exhibits a dispersion with the wavelength [2]. The change in refractive index ($\Delta n_{eff}$) may be caused by conditions of the surrounding environment such as temperature and analyte concentration. The bulk sensitivity S, one of the parameters for evaluating the biosensing performance, is defined as the change in the resonance wavelength $\Delta \lambda_{res}$ for a unit of the changed refractive index Δn of the cladding layer which is altered by the attached particles [38]:

(3) $$S\left(\frac{nm}{RIU}\right)=\frac{\Delta \lambda_{res}}{\Delta n}$$

To increase the sensitivity, the method of increasing the refractive index change amount has been developed. The utilization of a slot waveguide enables a change in refractive index due to a large light intensity by the overlapping of the leaked light from the slot and the attached analyte [4,26,38].

In this research, the mechanism of sensitivity enhancement by DSR with a long cavity can be explained by the enhanced area of the leak electric field intensity of the DSR waveguide. The DSR structures allow a larger area of the contact interfaces for the analyte to interact with electromagnetic field including the top interface and the side walls of the cavity. By extending the cavity length, it is able to use more contact surface for biomolecules to attach to the DSR cavities; hence, the change in resonance wavelengths increased to achieve an increased sensitivity.

Another important parameter improved by the DSR that impacts bio-sensitivity is the effect of the Q factor increment. The Q factor is an indicator for the peak sharpness required to obtain a precise resonance wavelength shift. The Q factor can be calculated using a function of the cavity perimeter L [39,40]:

(4) $$Q=\frac{{2\pi n}_{eff}}{\lambda_{res}}L.$$

Here, the light loss after a trip through the cavity is neglected. The Q factor shows a relation with the cavity length. The Q factor also depends on the structure of the cavity. In the biosensing application, cavity designs for Q factors of about 10^4^ are preferable for measurement ease and detection sensitivity. If the Q factor is too high, the wavelength tuning becomes difficult, and if the Q factor is too low, the shortened photon lifetime can reduce the analyte sensitivity.

Furthermore, the compact size of DSR designs takes advantage of biosensing a small amount of the analyte. In other words, the proposed DSR aims to design and fabricate a cavity structure for high-sensitivity and small-volume detection and easy fabrication. Firstly, the DSR cavity increases the bulk sensitivity and the Q factor due to the enhanced contact interface area between biomolecules and the evanescent field for a more significant resonance wavelength shift. Because of the extended cavity length, the area of the top and the side wall of the cavity waveguide, where the analyte interacts with the evanescent field to contribute to the resonance wavelength changes, is increased. Secondly, the DSR is designed in a micro-sized footprint to minimize the cavity size, to minimize the width of the cavity, thus reducing the amount of solution in a small fluidic channel for µL-level detection and helping the alignment procedure of the resonator into the microchannel to be easier.

In this research, the DSR cavities with about 1 mm cavity length combined with a thin-width polydimethylsiloxane (PDMS) microfluidic channel for detecting 1 µL of solution over the biosensor chip. The minimum microchannel sizes to contain 1 µL volume inside the microchannel are a length of 20 mm, a height of 200 µm, and a width of about 200 µm to 250 µm. The microchannel length was decided based on the chip size which is easy for handling and the size of the inlet and the outlet. The microchannel height was decided based on the fabrication mold of the microchannel. The cavity width is thus limited to be smaller than 50 µm for easing the alignment of the center PDMS microfluidic channel and the resonator. Hence, a cavity size of 40 µm × 90 µm was set to optimize the DSR design.

## 3. Simulation Analysis

**Figure 1:**
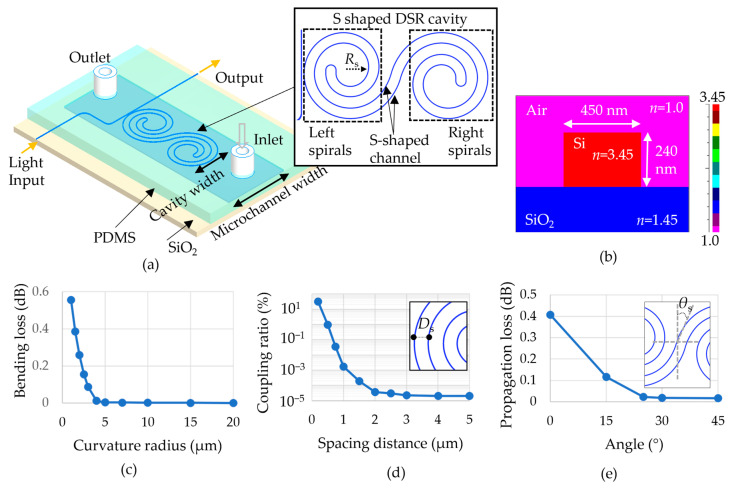
(**a**) Schematic of the double-spiral resonator (DSR) biosensor with a compact cavity size. The DSR is able to detect a small-volume analyte inside the thin-width microchannel. (**b**) Refractive index distribution of a waveguide cross-section. (**c**) Bending loss caused by the curvature radius. (**d**) Coupling ratio when the spacing distance (D_s_) changes. D_s_ is defined as the inset figure. (**e**) Power loss caused by the bending angles at S-shaped channel (θ_s_) as defined in the inset figure.

The DSR cavity was constructed by combining two single Archimedean spirals (left and right) and an S-shaped channel, as shown in Figure 1a. The spirals were designed to have the same spacing distance (D_S_) between successive turns to make good use of the cavity size. The right spiral was the vertically and horizontally flipped shape of the left spiral. An S-shaped channel waveguide linked two single spirals, for which θ_S_ is the angle between the vertical axis and the channel. Figure 1b shows a cross-section of the refractive index distribution of the Si-based DSR waveguide (refractive index, n_Si_: 3.45) built on a SiO_2_ substrate (n_SiO2_: 1.45), where air functions as the cladding layer (n_air_: 1). Different structures of the resonators were compared using an FDTD simulation (FullWAVE™ simulation tool, Synopsys) in the characteristic optimization of optical spectra to balance a high Q factor and the cavity length. DSR devices with optimized parameters using the simulation results were fabricated and their optical characteristics were analyzed. To save time in the simulation, a 2D FDTD analysis was used, in which the effective index of the waveguide was calculated using the finite element method (FEM) in a 3D waveguide structure with a width of 450 nm and a height of 240 nm [20].

There are three primary factors for designing a small-sized DSR, including the minimum radius R_s_ of the curvature, the spacing distance D_s_, and the shape of an S-shaped channel, to achieve a balance of low propagation loss and a high ratio of sensing surface to cavity size. During the simulation, the radiation loss and material scattering loss were neglected. Firstly, R_s_ was decided based on the bending loss caused by a bent waveguide’s curvature. Figure 1c shows a decreased bending loss depending on the curvature radius with a radius from 1 µm to 20 µm. It was noticed that an R_s_ with a value larger than 4 µm achieved minimal bending loss, which might provide a high Q factor; therefore, values of R_s_ ≥ 5 µm were used for designing the DSR cavity. Secondly, to examine D_s_, we simulated the dependence of the coupling ratio, which is the percentage of the light power of one waveguide to another waveguide at a spacing distance D_s_. Figure 1d indicates the coupling ratio from one waveguide to another waveguide in a spiral when the spacing distance changed from 0.2 µm to 5 µm. D_s_ ≥ 3 was selected to eliminate the light coupling to the other waveguides. Finally, optimizing the S-shaped channel for a low propagation loss was investigated by adjusting the angle θ_S_. Figure 1e indicates the propagation loss depending on the angle θ_S_ from 0 to 45°. The loss proportion was lower than 0.02 dB when θ_S_ ≥ 25°. Propagation loss occurs when mode-mismatched loss is caused by a bent channel for linking two spirals. Hence, an angle of 25° was utilized to achieve a low loss and minimize the cavity size to about 41 µm × 88 µm.

**Figure 2:**
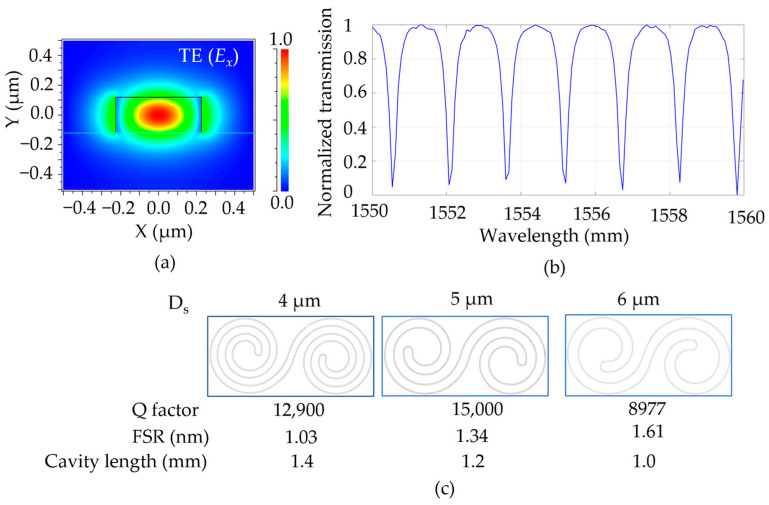
(**a**) Cross-section of the E field of a DSR waveguide with a width of 450 nm and a height of 240 nm in the TE (E_x_). (**b**) Simulated transmission spectrum for the DSR cavity with a spacing distance of 5 µm. (**c**) The images, Q factors, FSRs, and cavity lengths of different DSR designs with different spacing distances.

The transmission spectra for different DSR structures were studied using the FDTD method. The light was coupled to the DSR cavity via a bus waveguide placed at a gap distance apart from the cavity. Here, we designed a stripe-based bus waveguide with a width of 450 nm and a height of 240 nm, as large as the size of the DSR waveguide. The effective index was calculated using a finite-element-method (FEM) simulation. Figure 2a shows the electric field distribution (E_x_) of a DSR waveguide for the TE mode. A part of the leaked electric field over the waveguide at the top and side walls of the waveguide contributes to increasing attached biomolecules. A continuous wave is excited from the input of the bus waveguide, and the power intensity was monitored at the output. An example of the simulated transmission spectrum, for which the DSR had a cavity length of 1.2 mm, is shown in Figure 2b. The Q factor was calculated using a function of $\lambda_{res}/\lambda_{res\_FWHM}$, in which $\lambda_{res}$ is the resonance wavelength or the wavelength at the dip of the resonance, and $\lambda_{res\_FWHM}$ is the wavelength difference of the full width at half the maximum intensity. The free spectral range (FSR) is the distance between two dip wavelengths. Figure 2c shows the Q factors, FSR values, and cavity lengths for different spacing gaps (4, 5, and 6 µm) in the same cavity footprint of 41 µm × 88 µm. The DSR structure with a 5 µm spacing was selected for the fabrication because it could achieve a high Q factor and had a large sensing surface. It was noticed that, in principle, a larger cavity length might result in a higher Q factor; however, in a limited cavity size, the bending loss caused by the curvature radius could increase the bending loss, driving decreased Q factors. This is the reason that the DSR cavity with a 4 µm spacing distance had a smaller Q factor than that of the DSR cavity with a 5 µm spacing distance, even though it had a long cavity. Another reason that we selected the 5 µm spacing distance is that the FSR was long enough for analyzing the data. The FSR is reduced more when the cavity length is longer, which may cause some unrecognition of the changed dip wavelengths for minimal refractive index changes. The DSR with a 5 µm spacing distance and a cavity length of 1.2 mm balanced a large sensing area with a high Q factor.

**Figure 3:**
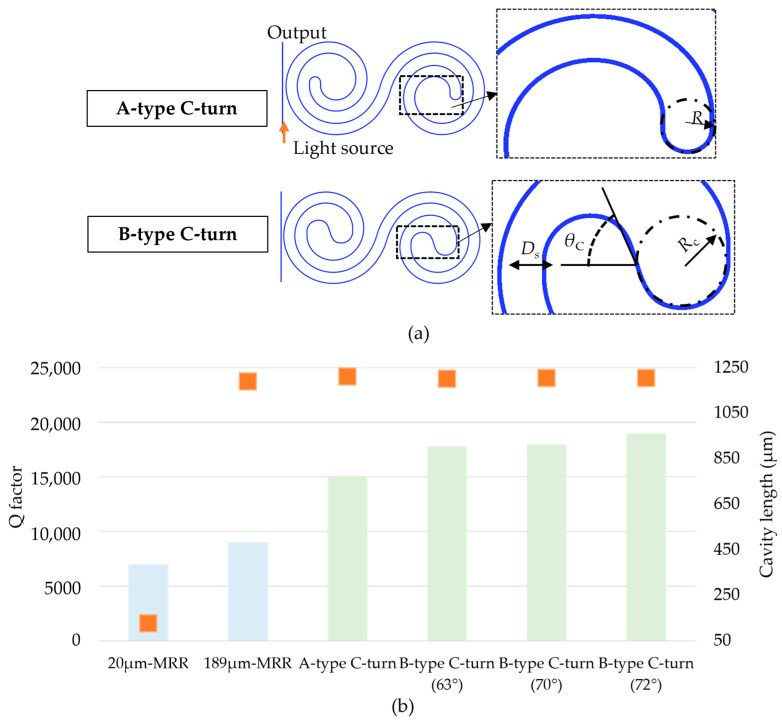
(**a**) Images of two different types of C-turns. The A-type C-turn had an arc diameter (R_c_) as large as the spacing distance D_s_. The B-type C-turn had two arcs with R_c_ = 5 µm linked to each other at an angle of θ_C_. (**b**) The column chart of Q factors (the left axis) for different cavities of conventional microring resonators (light blue) with R = 20 µm and 189 µm, and DSR cavities (light green) with different C-turn types. The orange square markers (the right axis) are the cavity lengths of the plotted cavities.

Finally, we designed the C-turn curve at the center of the spiral by calculating the Q factors of different types, as shown in Figure 3a. Here, we compared two types of the C-turns: the A-type, which is an arc with a diameter equal to the spacing distance (5 µm), and the B-type, which includes two arcs of a circle with a radius of 5 µm that join each other at an angle θ_c_. Figure 3b shows the column chart of the Q factors (the left axis) and cavity lengths (the right axis) for different types of resonators: MRR cavities with a radius of 20 µm and 189 µm, an A-type C-turn DSR cavity, and B-type C-turn DSR cavities with θ_c_ = 63°, 70°, or 72°. The 189 µm MRR has the same cavity length with DSR cavities. The 20 µm radius MRR has the same cavity width with the DSR cavities. The DSR designs allow the 10-time-enlarged contact interface in comparison with 20 µm radius MRR for the same cavity width. The DSR platform was reduced 680 times in comparison with 189 µm radius MRR, which has the same cavity length. The DSR structures (light green color) showed Q factors of 1.4–1.9 × 10^5^, which is two times higher than that of the MRR cavity with a radius of 20 µm, which had the same cavity width as the DSR structure. This is because the DSR cavity had a longer cavity length. We also found that the Q factors of the DSR structures were greater than that of the MRR cavity with a radius of 189 µm, which had the same cavity length as the DSR. This might be explained by the coupling ratio becoming larger when the light from the bus waveguide is coupled with a larger-radius ring so that the light power is conserved inside the MRR cavity, resulting in a smaller total Q factor. In addition, the B-type DSR expressed a higher Q factor than the A-type DSR. This increased Q factor was the result of less bending loss at the curvature in the B-type C-turn. We also recognized that the angle θ_c_ affected the Q factor values. The Q factor difference was because different bending curvatures can cause a mismatched mode of the propagating light. Eventually, DSR structures with a spacing gap of 5 µm and an angle θ_c_ of 72° were chosen for the fabrication.

Bulk sensitivity was simulated from the changed resonance wavelength when the attached surface or the top cladding surface had a different refractive index. The effective indices of the waveguide were calculated according to the changed refractive index of the top cladding surface. Based on the simulation results, a bulk sensitivity of 1500 nm/RIU was achieved for the DSR structure, while the bulk sensitivity of the 20 µm ring resonator was 150 nm/RIU. These results were compared with the experimental results. Furthermore, a surface sensitivity of 4100 pm/nm, calculated from the resonance wavelength shift for a unit thickness of the attached layer, was calculated. In this simulation, a model of an attached protein layer covering the DSR cavity was utilized [41]. The attached layer’s thickness needed to be less than the height of penetration depth. This simulated surface sensitivity was one order of magnitude higher than that of the 20 µm radius MRR. These results highlight the effect of enhancing the area of contact interface on the sensitivity by using DSR structures and the Q factors.

## 4. Experimental Methods

A 20 µm radius MRR, DSR cavities including the 1 mm length DSR, the 1.2 µm length DSR with an A-type C-turn, and the 1.2 µm length DSR with a B-type C-turn were fabricated on an SOI substrate (20 mm × 20 mm) with a 240 nm thick silicon layer on a 2.5 µm thick buried oxide (BOX) layer. The parallel waveguides at 250 µm apart were designed using Autodesk AutoCAD 2022 software. Taper-edged couplers were designed for the input and output facets, with the taper size reduced from 3 µm to 450 nm over a length of 30 µm. The length of the waveguides was 8 mm. The fabrication process included four steps of patterning by electron beam lithography (EBL), etching the silicon layer to transfer the waveguide patterns, cleaving the chip, and sticking the microfluidic channel. Firstly, the waveguides were patterned by EBL (ELS-G125S Elionix, Tokyo, Japan) using the 300 nm thick positive resist layer of ZEP520A (ZEON, Tokyo, Japan). Secondly, the pattern was transferred to the silicon layer by a dry etching process using deep reactive-ion etching (deep RIE) (MUC-21 ASE-SRE, Sumitomo Precision Products, Amagasaki, Japan) in CF_4_ and SF_6_ gas [42]. Thirdly, the device was cleaved at both sides of the input and output facet to achieve an 8 mm × 20 mm chip size. Herewith, the light from a laser source could be transmitted to the waveguide by placing a lensed fiber close to the 3 µm width facet of the taper-edged coupler. The resist layer could be removed in O_2_ plasma 100 W for 3 min (PDC210, Yamato Scientific Co., Ltd., Tokyo, Japan). Finally, a 20 mm thick PDMS film with a microfluidic channel was adhered to the sensor plate by heating the device with the PDMS film at 70° for 30 min. The microchannel had a width of 200 µm and a length of 16 mm. The PDMS film was placed at the position which overlapped the cavities. The alignment was achieved without observing under a microscope because the cavity width size was much thinner than the channel width. It was able to inject 1 µL volume solution into the microfluidic channel to contact with the resonator.

The sensing performances of the DSR cavities including the Q factors in air and water, the bulk sensitivities, and the intrinsic limits of detection were experimentally evaluated and compared with the 20 µm radius MRR. To clarify the sensing performances of the DSR cavity for different types of analytes using the microchannel-on-chip, experiments with acetone gas with different concentrations were demonstrated. The optical transmission spectra in various conditions were studied to quantify the sensing performances. The Q factors were calculated by dividing the resonance wavelength from the optical transmission spectrum by its full width at half maximum. The bulk sensitivity was estimated from the linear relationship between the refractive index of solutions with different concentrations and the resonance wavelength shifts. IPA solution (99.7%, Kanto Chemical Co., Tokyo, Japan) was used to measure bulk sensitivities. The solutions with different concentrations from 0.1% to 0.4% (*v*/*v*) were made by diluting IPA in deionized water. The intrinsic limit of detection defines a minimum refractive index change for shifting the resonance wavelength by a linewidth.

**Figure 4:**
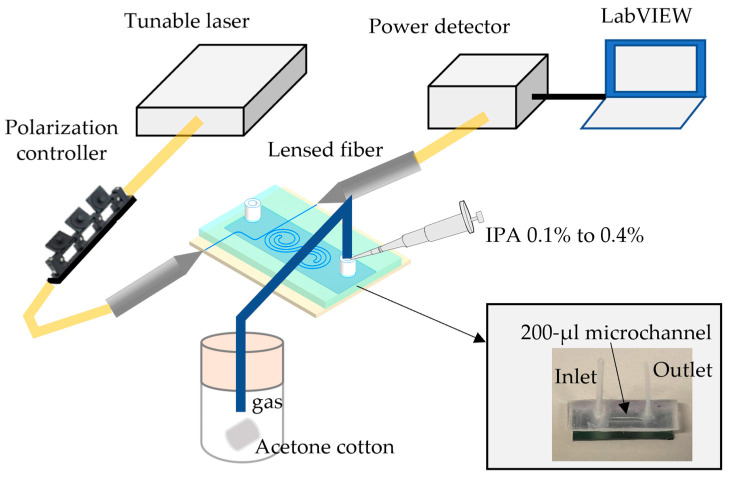
Schematic diagram of custom measurement setup. The PDMS microfluid channel adhered to the Si chip to make the microchannel. A micropipette was used to pump 1 µL of solution into the microchannel for detecting solution bulk sensitivity. In the VOC gas detection, acetone-infused cotton was placed inside a closed PFA bottle connected to the microfluidic inlet by a silicone tube, and the outlet was blocked.

Figure 4 shows the measurement setup for studying the optical transmission spectra at different conditions. The light (λ: 1.52–1.58 µm) was transmitted from a tunable laser (TSL-200, Santec Holdings Corporation, Komaki, Aichi, Japan). After the light passed through a 3-paddle polarization controller (FPC564, Thorlabs, Inc., Newton, NJ, USA), it was coupled with the taper coupler of the device via a lensed fiber. The output powers for various input wavelengths were collected by a power meter (PM101, Thorlabs, Inc., Newton, NJ, USA) measured at the lensed output fiber. The input wavelength and the output power were plotted with a LabVIEW program. The optical performance stability was evaluated by measuring resonance wavelength shifts for the same cavity over one hour at a room temperature of 25 °C. For measuring the optical characteristics in liquid, the 1 µL volume solutions were injected into the inlet using a micropipette. The measurement for the same solution was repeated every 2 min for 10 min. For measuring the acetone gas, a clean cloth permeated 50 µL of acetone (67-64-1, Kanto Chemical Co., Inc., Tokyo, Japan) was placed at the bottom of a closed 500 mL perfluoxy copolymer (PFA) bottle. A 10 cm long tube connected the PFA bottle to the chip inlet.

## 5. Experimental Results and Discussion

**Figure 5:**
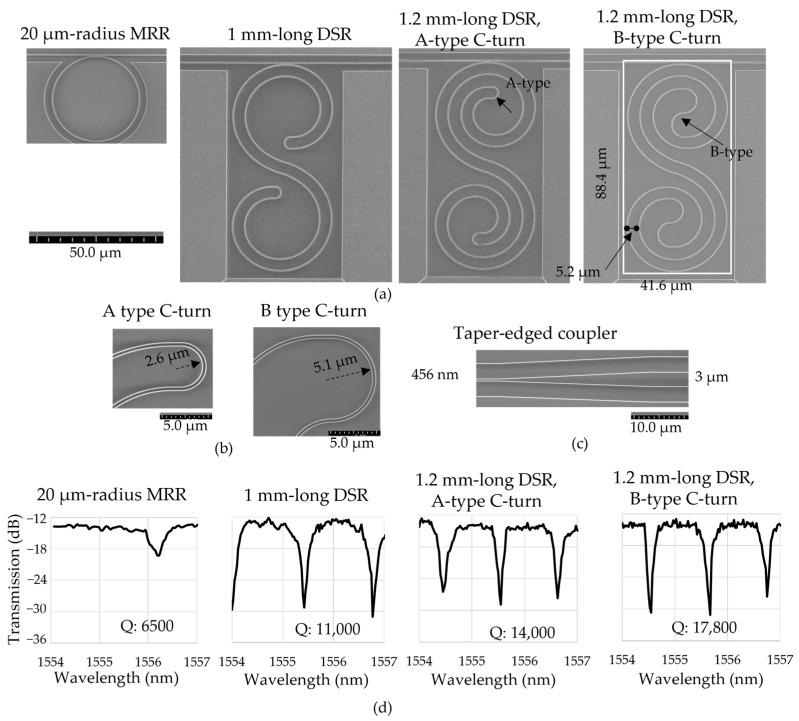
(**a**) SEM images of the 20 µm radius MRR, 1 mm length DSR, 1.2 mm length DSR (A-type C-turn), and 1.2 mm length DSR (B-type C-turn), starting from the left. The DSR cavity size was 41.6 µm × 88.4 µm. (**b**) SEM images of two types of C-turns. (**c**) SEM images of taper-edged coupler. (**d**) Transmission spectra in DI water for fabricated cavities with different cavity lengths.

Figure 5a shows the different types of cavities of the 20 µm radius MRR, the 1 mm length DSR, the 1.2 µm length DSR with an A-type C-turn, and the 1.2 µm length DSR with a B-type C-turn from left to right. The 20 µm radius MRR had a cavity size of 41 µm × 41 µm. The DSR cavity width was 41.6 µm, and the cavity length was 88.4 µm. The DSR footprint was reduced by 680 times compared to that of the 189 µm MRR, which had the same cavity length. The DSR cavities showed the advantages of reduced exposure-time in comparison with the MRR with the same cavity length due to the small scanning area of the EBL beam in a small platform.

The prototyped waveguide width was 456 ± 6 nm, while the designed width was 450 nm. The gap between the bus waveguide and the resonator was 290 ± 5 nm while the designed gap width was 300 nm. The waveguide size errors might have mostly occurred due to the EBL control of the resist fabrication. The reproducibility of the lithography process depends on the stability of EBL system, operator errors, and environmental conditions, such as the experimental temperature and the humidity, which affect the thickness and the affinity of the resist layer. The fabrication reproducibility using the EBL process can achieve the several-hundred-width waveguide with the error of sub 10 nm. However, the system stability might worsen the patterning process. Because the control of the waveguide width is important in the propagating mode loss, EBL process conditions for system stability such as the beam current and the aperture needed to be modified with a dummy sample before the main chip process. The optical power loss can also result from the roughness of the waveguide side walls and the edge coupler facet caused by the etching process. The deep RIE process with a slow etching rate of 18 nm/min was performed aiming to smooth the side walls. In addition, hydrogen annealing was reported to reduce the side wall roughness [43].

Figure 5b shows the SEM images of two different types of C-turns. The A-type C-turn had an arc diameter equal to the spacing distance, and the arc radius was 2.6 µm. The B-type C-turn had two arcs with a radius of 5.1 µm and a θ_C_ of 72°. The measured spacing distance was 5 µm, which is within the range required for the simulated spacing distance to prevent the coupling with the next curve. The taper-edged coupler had a length of 30 µm, changing from the waveguide with the size of 456 nm to the inserted facet with a size of 3 µm, as shown in Figure 5c.

The transmission spectra of the four types of cavities in air and DI water at a room temperature of 25 °C were collected. The resonance wavelength shift was about ±50 pm. The extinction ratio of the DSRs was 15 dB, while the extinction ratio of the MRR was 5 dB, measured in air. The measured propagation loss from straight waveguides was about −3.5 dB/cm, mainly from the scattering loss due to the roughness of the waveguide. From these resonance properties, the Q factors of the different designs in air and DI water were calculated. The Q factors in the air of the cavities of the 20 µm radius MRR, the 1 mm long DSR, the 1.2 mm long DSR with an A-type C-turn, and the 1.2 µm long DSR with a B-type C-turn were 0.73 × 10^4^, 1.29 × 10^4^, 1.55 × 10^4^, and 1.94 × 10^4^, respectively. Later, the transmission spectra in DI water were monitored using 1 µL of DI water inside the microchannel. Figure 5d shows the transmission spectra taken in DI water for 20 µm radius MRR, the 1 mm long DSR, the 1.2-mm long DSR (A-type C-turn), and the 1.2 mm long DSR (B-type C-turn), starting from the left. The Q factors in the DI water were reduced by 10% compared with those in the air due to the absorption loss in water. In DI water, the 1.2 mm long DSR with a B-type C-turn obtained the highest Q factor value of 1.78 × 10^4^. Consequently, with the same cavity width of 40 µm, the DSRs had Q factors that were two orders of magnitude higher than that of the 20 µm radius MRR in the air and water environments. Furthermore, the Q factor of the 1 mm long DSR cavity was higher than that of the MRR, but lower than that of the 1.2 mm long DSR. These results indicate that DSRs with a long cavity length can experimentally achieve a higher Q factor based on their cavity length. The shape at the C-turn is also a factor leading to an increased Q factor because it can affect the bending loss. The FSRs of the cavities of the 20 µm radius MRR, the 1 mm long DSR, the 1.2 mm long DSR with A-type C-turn, and the 1.2 mm long DSR with a B-type C-turn were 9.6 nm, 1.4 nm, 1.2 nm, and 1.2 nm, respectively. These results are close to the simulated results.

**Figure 6:**
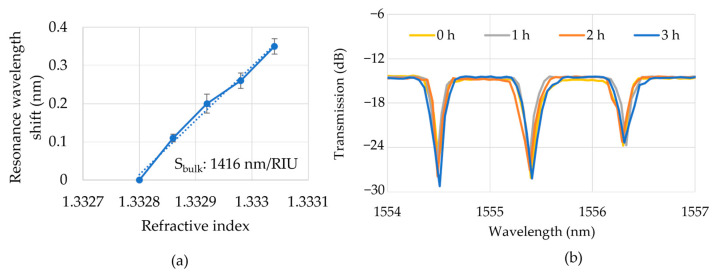
(**a**) Resonant wavelength shift for 1.2 mm length DSR cavity (B-type C-turn) over different refractive indices by using IPA solutions with concentrations of 0.1%, 0.2%, 0.3%, and 0.4%. (**b**) Measured transmission spectrum of IPA solution (0.1%) using DSR cavity at 0 h, 1 h, 2 h, and 3 h.

The 1.2 mm long DSR with a B-type C-turn was used to measure the bulk sensitivity in comparison with 20 µm radius MRR. Figure 6a shows the linear relationship between the refractive index (concentrations of 0.1% to 0.4%) of IPA solutions and the resonance wavelength shift of the 1.2 mm long DSR with a B-type C-turn. The refractive indices of IPA solutions were estimated from the linear relationship between the solution concentration and the refractive index [44]. We achieved a bulk sensitivity for the DSR of 1416 nm/RIU. The bulk sensitivity of the 20 µm radius MRR was 120 nm/RIU, as calculated by measuring the IPA solutions with concentrations of 1% to 3%. The selected IPA concentrations were different in the DSR cavity and MRR cavity to ensure the resonance wavelength shifted to be smaller than FSR. The bulk sensitivities of the 1.2 mm long DSR and 20 µm radius MRR in simulation were 1500 nm/RIU and 150 nm/RIU, respectively, as discussed in Section 3. Considering the reasons of the propagating loss caused by the fabrication process, the measurement system errors, and the surface hydrophilic, the experimental bulk sensitivity is very close to the simulation data. This result indicates the bulk sensitivity of the DSR was one order of magnitude larger than that of the 20 µm MRR in the simulation and experimental results as the result of the extended contact interface in the surface and the side wall of the DSR cavity. This result also indicates the highly exact simulation which can reduce much cost and time in fabrication. A standard deviation of the peak shift of ±20 pm was obtained. The peak shift of the same 1 µL IPA solution (concentration of 1%) was studied about ±50 pm, which was the result of the instability in the room temperature, the power and polarization matching of the input light from the tunable laser. Figure 6b shows the transmission spectrum of the 1 µL IPA solution at the times of 0 h, 1 h, 2 h, and 3 h. Therefore, the deviation of the peak shift in the bulk sensitivity measurement was in the range of the measurement condition error. In addition, it was able to keep the small-volume solution inside the microchannel for 3 h; even IPA is a volatile solution. Therefore, the effect of a longer cavity length increasing the bulk sensitivity was obvious in both the experimental results and the simulation, and using a compact cavity footprint combing with the microchannel could reduce the measurement solution.

**Figure 7:**
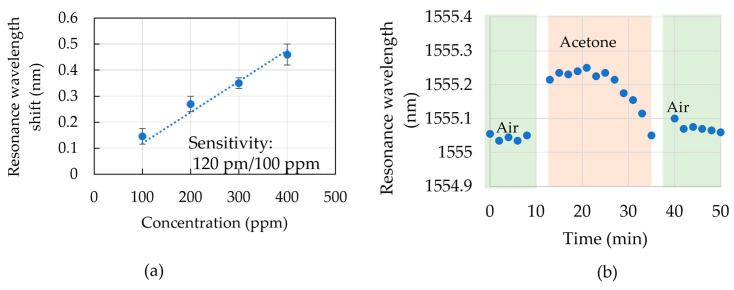
(**a**) Resonant wavelength shift for 1.2 mm length DSR cavity (B-type C-turn) over different acetone concentrations of 100 ppm, 200 pp, 300 ppm, and 400 ppm. (**b**) Resonant wavelength shifts as the environment inside the bottle changed in the sequence of air, acetone, and air. Acetone gas had a concentration of 100 ppm.

The 1.2 mm long DSR with a B-type C-turn was also used to detect acetone gas. Figure 7a shows resonance wavelength shifts when the acetone concentrations in the bottle were 100 ppm, 200 ppm, 300 ppm, and 400 ppm. The concentration sensitivity of 120 pm/100 ppm was calculated from the linear relationship between the resonance wavelength shift and the acetone concentration. A standard deviation of ±40 pm was obtained, which might result from the measurement environment. From this result, the detection limit of acetone concentration using the DSR cavity and microchannel fluidic was 100 ppm. Figure 7b shows the results of monitoring the resonance wavelength of acetone gas with a concentration of 100 ppm using a DSR cavity in a sequence of air, acetone gas, and air in real-time detection. The resonance wavelength shifted to 110 pm after the acetone gas appeared inside the bottle. A fast response in a resonance wavelength shift of several seconds was observed. After about 10 min of maintaining the same resonance wavelength shift, the resonance wavelength shift decreased gradually to the shift in the air. The wavelength shifts during the monitoring in the air and acetone conditions were in the range of the temperature shift. It is obvious that the acetone gas particles approached the cavity and interacted with the electric field over the cavity. However, it also vaporized through the PDSM film and attached to the side wall of the channel. Different materials, such as thermoplastic elastomers and soft thermoplastic elastomers, could be used instead of PDMS for the microchannel to obtain slow evaporation [45]. The DSR structure improved the sensitivity and quality factor for small-volume detection. The investigation using IPA solutions and acetone vapor indicated the variety of biosensing applications for measuring in different environments that are possible for label-free biosensing.

## 6. Challenges and Future Prospects

The S-shaped double-spiral resonator could reduce the analyte solution and enhance the refractive index sensitivity and the Q factor, which is the key characteristic evaluating the biosensing capability of evanescence-based biosensors. There were several challenges in the prototyped device fabrication method and measurement setup discussed. However, these issues might be overcome with the development of microfabrication in lab-on-chip technologies. One of the challenges of the DSR cavity for biosensing is the easy recognition of the wavelength shift due to the FSR length being shortened with the enhanced cavity length. A false reading out of the wavelength shift could occur if the wavelength shift by a large concentration were larger than the FSR. In the experiment, the real-time measurement of the transmission spectrum was studied to prevent the false reading-out. Hence, the DSR cavity length should be considered to optimize the target measurement concentrations. Another challenge of biosensing based on the refractive index changes is the selectivity to distinguish one from multiple attached biomolecules. This difficulty is because the effective refractive index would be defined by the total biomolecules attached to the contact interface to cause the resonance shift. The analyte types should be known as reference data for detection. To increase the selectivity, recent research of a serial microring resonator for detecting multiple analytes simultaneously was reported [46]. Different resonance wavelength MRRs with large FSR were designed based on hybrid plasmonic waveguides to recognize different solutions.

Further work will study the biosensing applications using the proposed DSR sensor. With the advantage of detecting a small amount of analyte inside the microfluidic, DSR can be applied for measuring a label-free detection of a low concentration of a protein analyte binding with target biomarkers by an optimized surface modification process [47,48]. Furthermore, the microchannel-on-sensor was helpful in preventing a small solution from evaporating. Another potential application of the DSR cavity is cell metabolism measurement in real time with high sensitivity because a greater portion of the cell membrane could interact with the evanescent field in a small footprint.

## 7. Conclusions

We demonstrated a novel structure of the S-shaped double-spiral resonator for an enhanced refractive index sensitivity to detect a small amount of analyte within a narrow-width microchannel. The DSR had a cavity length of about 1 mm and used dense spirals along the flow direction of the microfluidic channel on a compact footprint with a width of 40 µm, which could only contain a single 20 µm radius ring resonator with a cavity length of 125 µm. The DSR cavity with the enlarged cavity length resulted in a tenfold increase of the bulk sensitivity for different-concentration IPA solutions in comparison with the MRR with the same ring diameter of 40 µm due to the increased contact interface between the biomolecules and the waveguide leaky light. The Q factor of the DSR cavity with a cavity length of 1.2 mm, a cavity footprint of 41.6 µm × 88.4 µm, and a C-turn radius of 5.1 µm achieved a Q factor of ~1.9 × 10^4^, more than two times higher than that of the 20 µm radius MRR as the effect of the extended cavity length. A 1 µL solution was able to be kept inside the microfluidic for the measurement in 8 h with the resonance wavelength stability of ±5 pm. Real-time detection of acetone gas using the DSR cavity was achieved with a concentration sensitivity of 180 pm/100 ppm and a concentration detection limit of 100 ppm. The impact increase in the sensitivity, the Q factor, and small-volume detection in a small sensor footprint in real time are the impact advances for the biosensing development of an accurate, high-response, compact, and low-cost detection method of an ideal all-in-one optical biosensor.

## Data Availability

The data analyzed during the current study are available from the corresponding author upon reasonable request.

## Abbreviations

ELISA	Enzyme-linked immunosorbent assay
DSR	Double-spiral microresonator
SPR	Surface plasmon resonance
MZIs	Mach–Zehnder interferometers
CMOS	Complementary metal-oxide semiconductor
SOI	Silicon-on-insulator
MRR	Microring resonator
RI	Refractive index
FDTD	Finite-difference time-domain
IPA	Isopropyl alcohol
PDMS	Polydimethylsiloxane
FEM	Finite element method
FSR	Free spectral range
BOX	Buried oxide
EBL	Electron beam lithography
RIE	Reactive-ion etching
VOCs	Volatile organic compounds
